# Micro- and macroelement contents in the liver of farm and wild animals and the health risks involved in liver consumption

**DOI:** 10.1007/s10661-019-7274-x

**Published:** 2019-02-06

**Authors:** Alicja Kicińska, Paulina Glichowska, Magdalena Mamak

**Affiliations:** Faculty of Geology, Geophysics and Environmental Protection, AGH University of Science and Technology, Al. Mickiewicza 30, 30-059 Krakow, Poland

**Keywords:** Liver, Wild and farm animals, Macroelements, Microelements, Health risk assessment

## Abstract

The paper presents the macroelement (Al, Ca, Cu, Fe, K, Mg, Mn, Na, P, and Zn) and microelement (As, Cd, Co, Cr, Hg, Mo, Ni, Pb, and Sn) contents found in the liver of wild animals (boar and deer) and farm animals (rabbit, chicken, duck, cow, goat, and turkey). Statistically, the differences in element contents between the two groups were not significant (at *p* = 0.05), with the exception of Fe, K, Mg, Cd, Hg, Mo, and Pb. The liver of farm animals contained more Al, Cu, K, Mg, Na, Cr, and Sn, while the content of the remaining elements was higher in wild animals. An analysis of correlations between element content and age in wild animals (boar) showed that Pb and Al content increases with age, while Na and Cr contents decrease significantly. Comparisons between the test results and the maximum limits allowed by law showed that, in the case of wild animals, the regulatory limits were exceeded in 18% (for Cd and Cu) and 9% (for Hg) of the liver samples analyzed. In the case of farm animals, the limits for micro- and macroelement contents were not exceeded. The hazard index (HI) values for farm animals were lower than for wild animals, with regard to consumption by both children and adults. Based on the HI values calculated, it seems recommendable that consumption of the liver (preferably from farm animals) by children be limited to once weekly. For adults, the liver can be a valuable source of elements such as Zn, Fe, and Cr, which may be an indication for more frequent consumption.

## Introduction

The content of particular elements in various parts of the environment typically attracts much attention, and may even stir up emotion when viewed in the context of potential impact on living organisms, and especially on human health (Kicińska 2019). Health authorities pay particular attention to testing food produced by animal or plant farming for metal content. A number of research papers also discussed the issue of metal content in the tissues of wild animals (Bakowska et al. 2016, Dip et al. 2001, Długaszek and Kopczyński 2011, Medvedev 1999, Oyaro et al. 2007). Wild animals are commonly believed to be healthier, and their meat is safer for consumption, due to their “healthier,” more natural nutrition. Beside other factors, such as an animal’s age, health, or sex, the kind of food the animal eats is a particularly important factor affecting the accumulation of toxins (e.g., metals) in tissue. Researchers, including Neila et al. (2017), Damek-Poprawa and Sawicka-Kapusta (2004), Durkalec et al. (2015), Antonkiewicz et al. (2018), also demonstrated the importance of inter-species and individual differences resulting from adaptive processes related to inheritance of resistance in a given population or a given individual with increased resistance to high metal concentrations in the environment. Such resistance, involving the ability to exclude excess xenobiotics from metabolism or deposit them in specific organs, increases tolerance and changes the toxicity threshold (Kicińska 2016; Kicińska and Jelonek-Waliszewska 2017; Kołacz et al. 2003; Pietrzykowski et al. 2018). One such organ, especially with regard to metal accumulation, is the liver. It is a gland performing multiple functions (related to metabolism, circulation, and secretion), including the secretion of bile, which plays an important role in fat emulsification. The liver is involved in the synthesis and breakdown of glycogen; deamination and a range of amino acid transformations; synthesis of cholesterol, fatty acids, and phospholipids; and storage of blood and some vitamins and elements (e.g., Fe). It also contributes to the body’s defense mechanisms by removing and breaking down harmful substances from the bloodstream (Damek-Poprawa and Sawicka-Kapusta 2004).

The liver acts as a filter, or a chemical shield, that detoxifies the body by removing ingested toxins, physiological metabolites, or absorbed toxic chemicals. Its cells, called hepatocytes, absorb and deactivate dead, degenerated, or damaged cells that are no longer needed in the body, viruses and bacteria, and harmful substances ingested with food. These substances are neutralized by biochemical reactions and then excreted with bile. All kinds of the animal liver (chicken liver, cow liver, and others) are rich in minerals, including highly absorbable Fe, Cu, Zn, and Sn (Kabata-Pendias and Szteke 2012). It also has a high content of vitamins A, B2, B12, and C, folic acid, niacin, and pantothenic acid, greatly exceeding that found in any plant-based foods. Recommended values for human consumption vary depending on age and sex, ranging between 100 and 250 g of the animal liver per week (Burger 2007; Szkoda and Żmudzki 2002).

But is eating the liver really good for human health? We attempted to verify this by testing 33 liver samples from wild animals (*n* = 11) and farm animals (*n* = 22). The collected material was used to:

- determine the total content of the selected macroelements (Al, Ca, Cu, Fe, K, Mg, Mn, Na, P, Zn) and microelements (As, Cd, Co, Cr, Hg, Mo, Ni, Pb, Sn);
- identify differences in the content of these elements between wild and farm animals, as well as between the studied species (boar, deer, rabbit, chicken, cow, and goat);
- compare the test results with the regulatory limits for food and with data reported by other authors; and
- calculate the health risk involved in eating liver from both animal groups, associated with the content of selected elements (Al, As, Cd, Co, Cr, Cu, Fe, Hg, Mn, Mo, Ni, Pb, and Zn), for children and adults.

## Material and area of research

Study material was collected between May and September 2017 in the Lesser Poland Province (southern Poland, EU). Fresh liver samples, 100 g or more per sample, were obtained at two livestock buying stations. At the same time, surveys were used to collect information on the animals’ age, breeding and raising methods, and nutrition (for farm animals). For wild animals, the samples were obtained from three hunting clubs, and information on the animals’ age, sex, and body weight was provided by the hunters.

Fresh, raw liver samples were placed in plastic bags and frozen until completion of the material collection stage. The total number of samples was 33, including 11 samples from wild animals (10 boars, including 6 males and 4 females, and 1 deer). The animals were aged between 6 months and 5 years. Most material was collected from farm animals (*n* = 22 samples), including 11 chickens (mostly aged 1–2 months), 4 rabbits (3–12 months), 2 cows (2 and 18 months), 2 goats (2 and 36 months), 1 pig (6 months), 1 duck (8 months), and 1 turkey (18 months).

## Study methods

Raw samples were frozen and stored in plastic bags at − 80 °C. Once all the samples had been collected, they were defrosted and subsequently dried at 50 °C for 24 h (Adrian and Stevens 1979).

The dried material was ground using an electric grinder and treated with a mixture of concentrated acids (65% HNO_3_ and 35–38% HCl, 1:3) at a 1:10 ratio of solids to solution. Additionally, mineralization was performed using a 30% H_2_O_2_ solution at 105 °C for 2 h. The solution was then cooled and poured into test tubes. The concentrations of Al, As, Ca, Cd, Co, Cr, Cu, Fe, Hg, K, Mg, Mn, Mo, Na, Ni, P, Pb, S, Sn, and Zn in the extracts were determined by ICP-MS using an Elan 6100 system at the accredited AGH UST geo-hydrochemical laboratory in Kraków (certificate No. AB1050), with a precision of 10^−5^ mg/dm^3^. LOD and LOQ values were calculated using formulas presented in Kicińska (2018) and are shown in Tables 1 and 2. Repeatability of the results obtained was determined using an analysis of variance (FSP method—flexible statistical procedure—robust). Every 4th sample analyzed (the so called duplicate, their set comprised 25% of the population studied) was subject to distribution analysis and determination of element concentrations, just like the basic set of samples. The total variance and its components were as follows: δ^2^_total_ = 0.121, δ^2^_sampling_ = 0.006, δ^2^_analysis_ = 0.002, and δ^2^_technical_ = 0.008. In accordance with the method used (Ramsey et al. 1992), the obtained levels of uncertainty allow for making adequate chemical inferences. Study results were analyzed using the Statistica ver. 13.1 software.

**Table 1:** The total content of macroelements in the wild and farm animals’ liver (in mg kg^−1^ dry weight)

Animals (*n*)	Parameter	Al	Ca	Cu	Fe	K	Mg	Mn	Na	P	S	Zn
		(mg kg^−1^)										
QA	LOD	0.004	0.001	0.001	0.002	0.030	0.001	0.001	0.049	0.542	0.028	0.030
	LOQ	0.018	0.013	0.005	0.011	0.061	0.009	0.002	0.479	2.000	0.788	0.118
Wild (11)	Av	223	462	19	448*	6141*	452*	7	1761	16,172	10,334	91
	Min.–max.	35–596	340–721	8–62	129–717	5577–7116	356–490	3–10	1317–2490	13,707–18,139	8108–12,731	43–200
	SD	48	28	5	58	123	11	0.5	104	377	340	12
Farm (22)	Av	269	421	22	408*	6731*	498*	7	1943	16,236	10,780	70
	Min.–max.	18–1277	143–769	4–139	79–3144	4803–8076	419–596	3–12	1259–3248	13,223–20,216	8809–12,599	45–164
	SD	76	24	8	134	151	11	0.5	89	381	235	6
All (33)	Av	254	435	21	421	6534	483	7	1882	16,215	10,631	77
	Min.–max.	18–1277	144–769	4–139	79–3144	4803–8076	356–596	3–12	1259–3248	13,223–20,216	8108–12,731	43–200
	SD	53	19	5	91	119	9	0.4	70	284	197	6
	V	21	4	26	22	2	2	6	4	2	2	7
PL^1^		–	–	10(33)	–	–	–	–	–	–	–	150(499)
% of samples above PL:												
Wild Animals				18%								0%
Farm Animals				9%								0%

*n* no. of samples, SD standard deviation, *V* variability coefficient, *QA* quality of analyze, *LOD* limit of detection, *LOQ* limit of quantification, *–* lack data

^1^PL—permitted level (for wet weight, in bracket value transformed to dry weight) according to Regulation (2003)

*Statistical significant for *p* < 0.05

**Table 2:** The total content of microelements in the wild and farm animals’ liver (in mg kg^−1^ dry weight)

Animals (*n*)	Parameter	As	Cd	Co	Cr	Hg	Mo	Ni	Pb	Sn
		(mg kg^−1^)								
QA	LOD	0.0034	0.0022	0.0004	0.0007	0.0016	0.0015	0.0008	0.0015	0.0007
	LOQ	0.0073	0.0069	0.0021	0.0034	0.0048	0.0035	0.0027	0.0072	0.0024
Wild (11)	Av	0.16	1.15*	0.07	2.81	0.07*	2.67*	2.29	0.45*	1.35
	Min.–max.	0.07–0.56	0.25–4.02	0.01–0.12	2.34–2.99	0.02–0.40	1.37–4.61	1.86–3.09	0.08–1.01	1.13–1.63
	SD	0.04	0.30	0.01	0.06	0.03	0.25	0.11	0.09	0.04
Farm (22)	Av	0.10	0.34*	0.06	2.99	0.02*	2.04*	2.09	0.21*	1.44
	Min.–max.	0.04–0.29	0.03–0.89	0.01–0.12	2.39–8.07	0.01–0.05	1.32–2.88	1.81–2.81	0.08–0.65	1.23–1.77
	SD	0.01	0.06	0.01	0.25	0.002	0.10	0.05	0.03	0.03
All (33)	Av	0.18	0.61	0.06	2.93	0.04	2.25	2.16	0.59	1.41
	Min.–max.	0.04–0.56	0.03–4.02	0.01–0.12	2.34–8.07	0.01–0.40	1.32–4.61	1.81–3.09	0.08–1.01	1.13–1.77
	SD	0.02	0.12	0.01	0.17	0.01	0.12	0.05	0.04	0.03
	V	13	20	9	6	30	5	2	14	2
^1^PL		0.50(1.66)	0.50(1.66)	–	–	0.05(0.16)	–	–	0.50(1.61)	–
% of samples above PL:										
Wild Animals		0%	18%			9%			0%	
Farm Animals		0%	0%			0%			0%	

*n* no. of samples, *SD* standard deviation, *V* variability coefficient, *–* lack data

^1^PL—permitted level (for wet weight, in bracket value transformed to dry weight) according to Regulation (2003)

*Statistical significant for *p* < 0.05

To standardize the values published by other authors, we decided to report the test results in milligrams per kilogram of dry weight (DW), and fresh weight (FW) values reported in literature were converted to DW using formula (1) below, with an assumed moisture (water) content in the sample at 70%.

1 $$ {C}_{Me- md}={C}_{Me- mw}\bullet {\left(1-\frac{x}{100}\right)}^{-1}, $$

where:

C_Me-md_: content of the metal (Me) in a dry sample (mg kg^−1^)
C_Me-mw_: content of the metal (Me) in a fresh sample (mg kg^−1^)
*X*: moisture (water) content in the sample

To assess the potential health risk (HQ) for consumers, according to the US EPA methodology (2005, 2007), we used the determined concentration of selected elements found in the animal livers. Non-carcinogenic risk values were calculated using formula (2):

2 $$ \mathrm{HQ}=\frac{\mathrm{EDI}}{\mathrm{RfD}}, $$

where:

RfD: reference dose according to US EPA (2016)
EDI: estimated daily intake (mg kg^−1^ bw day^−1^), calculated using formula (3):

3 $$ \mathrm{ED}\mathrm{I}=\frac{\mathrm{C}\bullet \mathrm{EF}\bullet \mathrm{ED}\bullet {\mathrm{F}}_{\mathrm{IR}}}{{\mathrm{W}}_{\mathrm{A}\mathrm{B}}\bullet {\mathrm{T}}_{\mathrm{A}}}\bullet CF1, $$

where:

C: element content (mg kg^−1^)
EF: exposure frequency (365 or 52 days year^−1^)
ED: exposure duration (70 years for adults, 6 for children)
F_IR_: liver consumption (100 g person^−1^ day^−1^, calculated in mg)
W_AB_: average body weight (70 kg for adults, 20 kg for children) (US EPA 2011)
T_A_: average exposure time (365∙ED), while in the case of carcinogens the value is 25,550 (days)
CF1: unit conversion factor of 10^−6^

Hazard index (HI) was calculated as the sum of HQ values. At HI ≤ 1, the probability of adverse health effects is low, at HI *>* 1, adverse effects are probable, while values of HI > 10 indicate high exposure and high chronic health risk associated with toxicity. To establish a level of the cancer *Risk* for carcinogens, the HQ doses were multiplied by the respective slope factors (US EPA 2005, for Pb WHO 1993).

## Results and discussion

### Macroelement content in the liver of wild and farm animals

Out of all elements tested for, P and S dominated in the chemical composition, with values of 13,223–20,216 mg kg^−1^ DW (dry weight) for P and 8108–12,731 mg kg^−1^ DW for S (Table 1). Considerable levels of K, Na, and Al were also found, with contents of 4803–8076, 1259–3248, and 18–1277 (mg kg^−1^ DW), respectively. As to highly absorbable elements, which included Fe, Ca, Cu, Mg, and Zn, the respective contents were 79–3144, 144–769, 4–139, 356–596, and 43–200 mg kg^−1^ DW. Small amounts of Mn were also found (3–12 mg kg^−1^ DW).

**Fig. 1:**
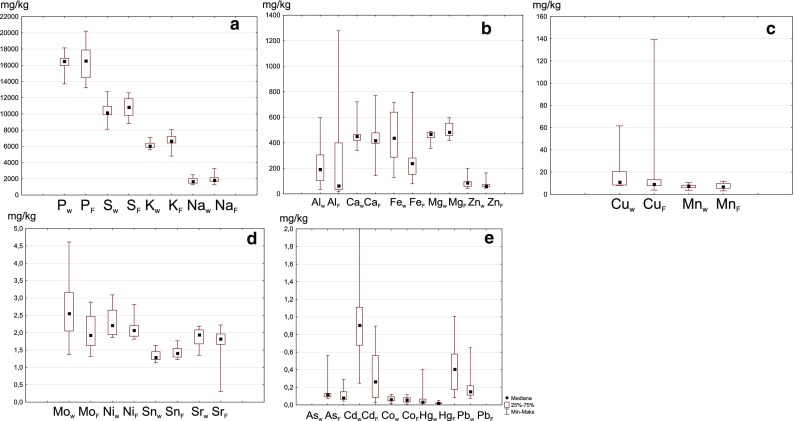
Statistical diversity of content of macro- (**a-c**) and microelements (**d-e**) in the livers of wild (W) and farmed (F) animals

A comparison of mean macroelement content found in the livers of wild and farm animals showed no significant differences (Fig. 1a–c). Samples from farm animals were found to contain more Al (by 21% on average), Cu (16%), and K, Mg, and Na (10%). Mn and P levels were found to be nearly identical in these two animal groups. Slightly lower results were obtained for farm animals than for wild animals with regard to content of Zn (23% lower) and Ca and Fe (approx. 9% lower). However, in the samples from farm animals, the dispersion of values was considerably larger (Fig. 1). The differences in macroelement content between wild and farm animal liver were not statistically significant (at *p* = 0.05), except in the case of Fe, K, and Mg. In the studied population of wild animals, the respective levels of these elements were 448, 6141, and 1761 mg kg^−1^ DW, while in the population of farm animals, the respective values were 408, 6731, and 498 mg kg^−1^ DW (Table 1).

### Microelement content in the liver of wild and farm animals

The microelement group included, As, Cd, Co, Cr, Hg, Mo, Ni, Pb, and Sn, and the content of these elements in the samples was 0.04–0.56, 0.03–4.02, 0.01–0.12, 2.34–8.07, 0.01–0.40, 1.2–4.61, 1.81–3.09, 0.08–1.01, and 1.13–1.77 mg kg^−1^ DW, respectively (Table 2). For wild animals, the mean levels were as follows (in mg kg^−1^ DW): As 0.16, Cd 1.15, Co 0.07, Cr 2.81, Hg 0.07, Mo 2.67, Ni 2.29, Pb 0.45, and Sn 1.35. For farm animals, the calculated means were slightly higher in two cases (values in mg kg^−1^ DW): for Cr the mean was 2.99 (i.e., approx. 6% higher than for wild animals), and for Sn, it was 1.44 (approx. 7% higher). The mean levels of the remaining elements were between 4 and 70% lower than in wild animals. The values (in mg kg^−1^ DW) were as follows: As 0.10, Cd 0.34, Co 0.06, Hg 0.02, Mo 2.04, Ni 2.09, and Pb 0.21 (Fig. 1d, e). Statistically significant differences (at *p* = 0.05) were only found for Cd, Hg, Mo, and Pb.

The highest variability in the entire material was found for the concentrations of Hg (V = 30), Cu (V = 26), Fe (V = 22), Al (V = 21), and Cd (V = 20). In this group, the so-called extreme values were also found (Fig. 1).

### Species-, age-, and sex-related differences in chemical composition

Though the studied animal populations were not large, an attempt was made to analyze differences in the chemical composition of the liver in terms of the species, age, and sex of the animals.

#### Wild animals

In the wild animal group, the boar liver contained more Al, Ca, Cd, Co, Cr, Fe, Ni, Pb, and Zn than the deer liver. The mean levels of these elements were 224.3, 465.4, 1.17, 0.06, 2.82, 480, 2.32, 0.49, and 95.3 mg kg^−1^ DW, respectively, in the boar liver, and 208.8, 430.8, 0.91, 2.75, 129, 1.95, 0.08, and 49.11 mg kg^−1^ DW, respectively, in the deer liver. The deer liver contained more As (0.92 mg kg^−1^ DW), Co (0.10 mg kg^−1^ DW), and Cu (61.60 mg kg^−1^ DW)—in the boar liver, the levels of these elements were 0.16, 0.06, and 14.48 mg kg^−1^ DW, respectively. Mg and Mn contents were similar between the two species (Table 3).

**Table 3:** Concentration of elements in the liver of various species of wild animals (in mg kg^−1^ dry weight)

Animal	Parameter	Al	As	Ca	Cd	Co	Cr	Cu	Fe	Mg	Mn	Ni	Pb	Zn
		(mg kg^−1^)												
Wild boar (*S. scrofa*)	Range^1^	–	–	–	udl–4.52	–	–	9.16–63.26	–	–	–	–	0.01–4.39	7.25–235.3
	Av ± SD^1^				0.33 ± 0.53			23.50 ± 9.71					0.38 ± 0.56	56.86 ± 39.04
	Range^2^	–	–	–	0.17–1.50	–	–	nd	nd	–	–	nd	0.57–3.23	nd
	Av ± SD^2^				0.53 ± 0.13								1.53 ± 0.27	
	Range^4^	2.76–22.91	0.09–0.11	–	–	–	–	–	–	–	–	–	–	–
	Range^5^	2.50–4.93	–	166–700	0.50–2.53	–	0.10–2.40	8.16–19.85	553–1508	439–679	2.50–6.33	0.09–2.86	0.37–1.43	90.2–330.7
	Av ± SD^5^	3.60 ± 1.03		298 ± 196	1.10 ± 0.60		0.47 ± 0.70	12.82 ± 3.46	866 ± 333	539 ± 83	4.16 ± 1.16	0.50 ± 0.83	0.83 ± 0.27	134.7 ± 70.9
	Range^6^	35.0–596.0	0.075–0.561	340–721	0.25–4.02	0.01–0.12	2.34–2.98	8.03–43.95	209–717	356–490	3.51–10.48	1.86–3.09	0.14–1.01	43.4–200.2
	Av ± SD^6^	224.3 ± 52	0.163 ± 0.042	465 ± 30	1.17 ± 0.32	0.06 ± 0.01	2.82 ± 0.06	14.48 ± 3.26	480 ± 54	451 ± 12	7.24 ± 0.60	2.32 ± 0.12	0.49 ± 0.09	95.3 ± 12.3
White-tailed deer (*O. virginianus*)	Range^3^	–	–	–	–	–	–	–	–	–	–	–	–	–
	Av ± SD^3^				0.70 ± 2.7	0.27 ± 0.09		107.49 ± 12.72				1.66 ± 0.50		166.27 ± 24.44
Roe deer (*C. capreolus*)	Range^4^	1.36–7.33	0.040–0.196	–	0.93–1.06	–	–	70.39–72.76	–	–	–	–	0.53–1.16	81.21–81.38
	Range^5^	0.43–3.79	–	124–907	0.33–7.96	–	0.09–0.23	7.26–188.64	146–1958	483–1029	2.59–12.12	0.03–1.06	0.96–3.43	80.6–171.5
	Av ± SD^5^	2.53 ± 1.19		283 ± 222	2.63 ± 2.29		0.13 ± 0.03	87.18 ± 63.6	683 ± 456	569 ± 136	7.49 ± 2.43	0.27 ± 0.30	1.43 ± 0.60	116.5 ± 30.9
	Av^6^	208.8	0.92	430.8	0.91	0.10	2.75	61.60	129	450	7.68	1.95	0.08	49.11

*nd* not detected, *udl* under detection limit, *–* lack data

^1^Neila et al. (2017)/Spain (dry weight)

^2^Medvedev (1999)/Russia (wet weight calculated to dry weight)

^3^Khan et al. (1995)/USA (wet weight calculated to dry weight)

^4^Kucharczak and Moryl (2012)/Poland (wet weight calculated to dry weight)

^5^Długaszek and Kopczyński (2011)/Poland (wet weight calculated to dry weight)

^6^This study (2017)/Poland (dry weight)

Comparisons between the obtained results and values reported by other authors show that:

- the obtained results for Al, Ca, Cd, Cr, Cu, Fe, Mg, Mn, Ni, Pb, and Zn levels are considerably higher than those reported by Długaszek and Kopczyński (2011) in Polish boars, and the obtained As and Al levels are considerably higher than those reported by Kucharczak and Moryl (2012) in the same population;
- the obtained Cd levels are slightly higher than those reported by Medvedev (1999), who studied the livers of Russian boars, while Pb results reported by the author were very similar to those found in the present study;
- the obtained results for Cd, Cu, Pb, and Zn were similar to those reported by Neila et al. (2017) in the boar liver. For Cd, Pb, and Zn, the mean levels reported by the author were 0.33, 0.38, and 56.86 mg kg^−1^ DW, respectively—slightly lower than those found in the present study: 1.17, 0.49, and 95.3 mg kg^−1^ DW, respectively. For Cu, the opposite was found in the Spanish samples analyzed by Neila et al. (2017); the mean content of this metal was 23.50 mg kg^−1^ DW (range 9.16–63.26 mg kg^−1^ DW), while in the present study, the mean Cu content was 14.48 mg∙kg^−1^ DW, and the range was 8.03–43.95 mg kg^−1^ DW; and
- with regard to the deer liver, we found considerably higher levels of Al, As, Ca, Cr, Mn, and Ni; comparable levels of Cd, Co, and Mn; and considerably lower levels of Cu, Fe, Mg, Pb, and Zn than those reported for the deer liver samples by Khan et al. (1995) and Długaszek and Kopczyński (2011) (Table 3).

In the boar population, samples were collected both from males and females. The calculated means and standard deviations indicate that the levels of Al, As, Hg, K, Mg, Pb, and Zn are higher in male boars (Table 4). The livers of female boars contained approx. 5–10% less Al, K, and Mg and approx. 30–40% less As, Pb, Zn, and Hg. On the other hand, the livers of female boars contained approx. 5–7% more Cr, Na, and Sn; approx. 10–20% more Ca, Fe, Mo, Ni, Mn, and S; approx. 40% more Co; and over 100% more Cu and 150% more Cd. P concentrations were nearly identical in both sexes. Very high variability was found for Al and Hg in males and for Cd and Cu in females. Similar findings were reported by Khan et al. (1995) in their analysis of levels of Cd and other heavy metals in the liver of white-tailed deer in Alabama. They found higher Cu and Zn levels in females and higher Cd, Co, and Ni levels in males. In a study of boars living in Galicia (NW Spain), Neila et al. (2017) found higher Zn, Cd, and Pb contents in the liver samples from males (55.78, 0.346, and 0.424 mg kg^−1^ DW, respectively), compared to samples from females (45.25, 0.305, and 0.341 mg kg^−1^ DW, respectively). The opposite was found for Cu, with slightly higher concentration of this metal found in female boars (23.83 mg kg^−1^ DW) than in males (23.08 mg kg^−1^ DW).

**Table 4:** Average contents (mg kg^−1^ dry weight) and standard deviations of elements calculated for the livers taken from female and male of wild boar species

Element	Arithmetic mean ± standard deviation	
	Male specimens (*n* = 6)	Female specimens (*n* = 4)
Al	229.3 ± 85.5	216.9 ± 30.5
As	0.18 ± 0.07	0.13 ± 0.01
Ca	425.9 ± 22.2	524.5 ± 56.7
Cd	0.74 ± 0.13	1.81 ± 0.67
Co	0.05 ± 0.006	0.08 ± 0.02
Cr	2.74 ± 0.09	2.93 ± 0.02
Cu	10.27 ± 0.80	20.79 ± 7.11
Fe	435.1 ± 71.6	546.9 ± 74.0
Hg	0.09 ± 0.05	0.06 ± 0.02
K	6311 ± 160	5822 ± 133
Mg	457.5 ± 18.7	442.9 ± 12.5
Mn	6.80 ± 0.9	7.90 ± 0.51
Mo	2.47 ± 0.13	2.87 ± 0.63
Na	1756 ± 174	1841 ± 81
Ni	2.22 ± 0.11	2.48 ± 0.22
P	16,066 ± 443	16,054 ± 743
Pb	0.56 ± 0.14	0.39 ± 0.06
S	9930 ± 426	10,787 ± 576
Sn	1.32 ± 0.04	1.40 ± 0.09
Zn	111.4 ± 16.6	71.1 ± 10.4

*n* number of sample

With a significance threshold set at *p* = 0.05, the calculated differences in levels of the analyzed elements between the livers from male and female boars were not significant.

Correlation coefficients were calculated for element content and animal age (at three significance thresholds: *p* = 0.05, *p* = 0.01, and *p* = 0.001) in the wild animal population (Table 5). High correlation (0.5 ≤ *r* < 0.7) was found for the following combinations: Mg–K, Mg–S, S–Mn, Fe–Cd, Cd–P, Co–Cu, Cr–Al, Cr–Na, Ni–Na, Pb–Na, Pb–As, and Pb–Hg. Similarly high inverse correlation was found for Na–Al, Cu–Fe, Zn–Cu, and Pb–Al. Very high correlation (0.7 ≤ *r* < 0.9) was found for P–Mg, Mn–Al, Zn–K, Mo–Cd, and Ni–Ca. Almost full correlation (0.9 ≤ *r* < 1) was only found for the Hg–As pair.

**Table 5:** Correlation coefficients (*r*_*xy*_) between the marked elements’ contents in the livers of wild animals and age

Factor	Al	Ca	Fe	K	Mg	Na	P	S	Cu	Mn	Zn	As	Cd	Co	Cr	Hg	Mo	Ni	Pb	Sn
Age	0.62*	− 0.14	0.20	0.03	0.07	− 0.84**	0.16	0.09	0.32	0.34	0.40	0.11	0.09	0.09	− 0.65*	0.09	0.43	0.20	*0.57*	0.03
Al	–	− 0.03	− 0.07	0.02	0.30	*− 0.51*	0.30	0.30	0.00	0.74**	− 0.16	− 0.19	0.03	0.14	*0.56*	− 0.19	0.23	0.10	*− 0.51*	0.10
Ca		–	− 0.22	− 0.19	0.01	0.40	− 0.24	0.28	0.41	0.45	− 0.17	− 0.03	0.03	0.35	0.24	− 0.08	− 0.36	0.81**	0.27	− 0.13
Fe			–	− 0.27	− 0.27	− 0.25	− 0.13	− 0.30	*− 0.54*	− 0.33	− 0.05	0.01	*0.52*	− 0.38	0.42	0.07	0.45	− 0.22	− 0.09	0.31
K				–	*0.55*	0.20	0.48	0.22	− 0.16	− 0.18	0.80**	− 0.10	0.08	− 0.29	− 0.27	− 0.14	0.20	− 0.05	0.24	0.19
Mg					–	0.26	0.85**	*0.59*	− 0.25	0.41	0.39	0.24	0.31	0.14	− 0.26	0.21	0.32	0.01	0.38	0.39
Na						–	0.01	0.32	− 0.15	− 0.22	0.46	− 0.08	0.12	0.14	*− 0.56*	− 0.10	− 0.38	*0.50*	*0.56*	0.12
P							–	0.69*	− 0.10	0.34	0.21	0.20	*0.54*	0.24	− 0.08	0.22	0.67*	− 0.22	0.02	0.47
S								–	*0.12*	*0.58*	0.09	− 0.03	0.60*	0.37	0.07	− 0.03	0.45	0.25	− 0.12	0.09
Cu									–	0.29	*− 0.58*	− 0.20	− 0.21	*0.57*	0.11	− 0.22	− 0.18	0.18	− 0.34	− 0.30
Mn										–	− 0.37	0.13	0.09	0.45	0.44	0.10	0.12	0.35	− 0.19	− 0.07
Zn											–	− 0.01	0.03	*− 0.51*	− 0.30	− 0.05	− 0.01	0.11	0.45	0.26
As												–	− 0.11	0.18	− 0.08	0.99***	0.02	− 0.04	*0.60*	− 0.01
Cd													–	− 0.01	0.23	− 0.06	0.81**	− 0.10	− 0.16	0.31
Co														–	0.03	0.21	− 0.18	0.43	− 0.07	0.15
Cr															–	− 0.07	0.33	0.28	− 0.49	0.18
Hg																–	0.06	− 0.08	*0.55*	0.01
Mo																	–	*− 0.50*	− 0.35	0.26
Ni																		–	0.24	0.12
Pb																			–	0.08
Sn																				–

High correlation (0.5 ≤ *r* < 0.7) (italics); very high correlation (0.7 ≤ *r* < 0.9) (boldface); almost full correlation (0.9 ≤ *r* < 1) (bold italics)

*p < 0.05; **p < 0.01; ***p < 0.001 statistically significant

The following correlations were found to be statistically significant: S–P, Cd–S, and Mo–P at *p* = 0.05; P–Mg, Mn–Al, Zn–K, Mo–Cd, and Ni–Fe at *p* = 0.01; and Hg–As at *p* = 0.001.

An analysis of correlations between element content and age in wild animals showed that Pb (*r* = 0.57) and Al (*r* = 0.62) contents increase with age, while Na (*r* = − 0.84) and Cr (*r* = − 0.65) contents decrease significantly. For the remaining elements, no statistically significant correlations were found. Similar findings were reported by Khan et al. (1995), who found a statistically significant correlation between age and Ni content only and no significant correlation (at *p* < 0.05) for Cd, Co, Cu, and Zn.

#### Farm animals

**Fig. 2:**
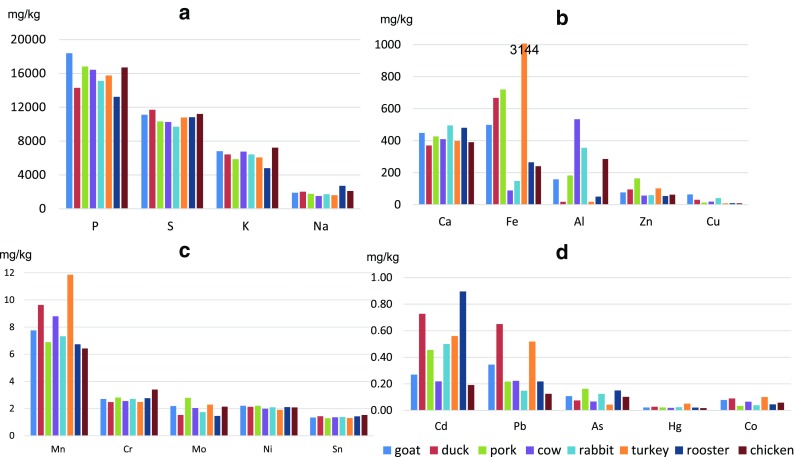
Average content of macro- (**a-b**) and microelements (**c-d**) in the livers of farmed animals

The other, much larger, group studied included farm animals, and specifically, chicken, duck, goat, turkey, rabbit, and cow (calf). Comparisons for such a large number of elements are rather difficult, mainly due to the wide variance in their concentrations. Therefore, the analyzed elements were divided into four groups (Fig. 2). Group 1 included P, S, K, and Na (Fig. 2a). Their presence in animal tissue is obvious, and these were the dominant elements out of all analyzed in the present study (Table 1). The highest P content was found in the goat liver (mean 18,393 mg kg^−1^ DW), with slightly lower values in the pig (16,807 mg kg^−1^ DW) and chicken livers (16,702 mg kg^−1^ DW). The lowest P content was found in the rooster liver (13,223 mg kg^−1^ DW). These results are considerably higher than the P content listed in the basic report from USDA (ndb.nal.usda.gov). For S, the highest levels were found in the duck liver (11,697 mg kg^−1^ DW), the lowest in the rabbit liver (9698 mg kg^−1^ DW) which also contained the least K (4,803 mg kg^−1^ DW). The highest K content was found in the chicken liver (7214 mg kg^−1^ DW). Na levels were the highest (2709 mg kg^−1^ DW) in the rooster liver and the lowest (1506 mg kg^−1^ DW) in the cow liver.

Group 2 included the elements required for normal growth and functioning of the animal body: Ca, Fe, Al, Zn, and Cu (Fig. 2b). The highest levels of these elements were found (respectively, in mg kg^−1^ DW) in the following: rabbit (495), turkey (3144!!), cow (534), pig (164), and goat livers (64). The lowest Ca, Fe, Al, Zn, and Cu levels were found (respectively, in mg kg^−1^) in the following: duck (370), cow (89), turkey (18), rooster (54), and chicken livers (8.6). The Ca values found in the duck, pig, cow, and chicken livers are considerably higher than those published by the USDA (ndb.nal.usda.gov). The Cu levels found in the present study are considerably higher than those reported by Oyaro et al. (2007) in Kenyan cattle, but very similar to those found by Kołacz et al. (2003) in chickens form Polish farms (Table 6). A significant difference was found for Fe. We found a mean Fe level of 89 mg kg^−1^ DW in the cow liver, whereas the US agency published a value of 65 mg kg^−1^ FW or 218 mg kg^−1^ after conversion to DW. Much smaller differences were found for the chicken liver: in the present study, we found a mean Fe content of 240 mg kg^−1^ DW in these samples, while the USDA reports a value of 116 mg kg^−1^ FW or 387 mg kg^−1^ DW. The Zn levels we found in the duck and pig livers were nearly identical to those reported in the database available at ndb.nal.usda.gov. For the cow and chicken livers, our results were lower (ndb.nal.usda.gov, Kołacz et al. 2003).

**Table 6:** Concentration of elements in the liver of various species of farm animals (in mg kg^−1^ dry weight)

Animal		Parameter	Al	As	Ca	Cd	Cu	Fe	Hg	K	Mg	Na	P	Pb	Zn
			(mg kg^−1^)												
Goat (*Capra hircus*)		range^1^	22–295	0.062–0.152	417–479	0.062–0.478	3.8–125.1	200–796	0.019–0.026	6540–7078	455–564	1705–2066	16,570–20,216	0.157–0.533	72.55–79.87
		Av ± SD^1^	159 ± 97	0.107 ± 0.032	448 ± 22	0.270 ± 0.149	64.5 ± 43.3	498 ± 213	0.022 ± 0.002	6809 ± 192	510 ± 39	1885 ± 129	18,393 ± 1302	0.345 ± 0.134	76.21 ± 3.6
Duck (*Anas platyrhynchos* f. *domestica*)		Av^1^	18	0.075	370	0.727	30.86	667	0.028	6414	467	2012	14,293	0.650	95.30
		Av^2^	–	–	366	–	–	1017	–	7659	799	4662	8958	–	102.2
Pork (*Sus scrofa* f. *domestica*)		Av^1^	182	0.163	426	0.455	13.15	720	0.022	5852	458	1755	16,807	0.217	163.89
		Av^2^	–	–	300	–	–	776	–	9091	599	2897	9590	–	191.8
		Av^6^	–	0.056	–	0.20	–	–	0.007	–	–	–	–	0.23	–
Cow (*Bos taurus taurus*)		Range^1^	35–1033	0.064–0.069	402–416	0.164–0.273	18.13–20.46	78.9–98.9	0.019–0.020	6292–7219	458–159	1258–1753	16,098–16,759	0.174–0.271	47.10–65.54
		Av ± SD^1^	534 ± 356	0.067 ± 0.002	409 ± 5	0.219 ± 0.039	19.30 ± 0.83	88.9 ± 7.1	0.019 ± 0.001	6756 ± 331	459 ± 0.1	1506 ± 176	16,428 ± 236	0.222 ± 0.035	56.32 ± 6.58
		Av^2^	–	–	200	–	–	218	–	11,722	699	2631	16,550	–	176.5
		Range^4^	–	–	–	0.003–0.009	0.07–4.75	–	–	–	–	–	–	0.16–0.17	1.26–4.27
		Av ± SD^4^	–	–	–	0.005 ± 0.002	1.99 ± 1.33	–	–	–	–	–	–	0.17 ± 0.003	1.97 ± 0.80
		Av^6^	–	0.063	–	0.43	–	–	0.009	–	–	–	–	0.33	–
Rabbit (*Oryctolagus cuniculus* f. *domesticus*)		Range^1^	33–909	0.075–0.187	396–769	0.156–0.677	5.75–139.23	131–159	0.020–0.028	5813–6791	440–489	1474–1995	13,515–16,529	0.111–0.219	45.31–65.09
		Av ± SD^1^	355 ± 175	0.124 ± 0.025	495 ± 79	0.500 ± 0.104	40.93 ± 28.4	148 ± 5	0.024 ± 0.001	6415 ± 182	467 ± 9	1717 ± 98	15,114 ± 538	0.148 ± 0.022	58.94 ± 4.0
		Range^5^	–	–	–	0.13–2.33	–	–	–	–	–	–	–	0.30–2.23	–
		Av^6^	–	0.063	–	0.43	–	–	0.007	–	–	–	–	0.23	–
Turkey (*Meleagris gallopavo gallopavo* var. *domesticus*)		Av^1^	18	0.043	399	0.560	8.64	3144	0.051	6069	486	1603	15,751	0.518	101.95
Poultry (*Gallus gallus domesticus*)	Rooster	Av^1^	50	0.150	480	0.896	9.20	265	0.021	4803	478	2709	13,223	0.217	54.24
	Chicken	Range^1^	28–1277	0.053–0.291	144–526	0.026–0.627	6.90–10.17	153–295	0.015–0.024	6462–8076	419–596	1638–3248	14,097–18,450	0.077–0.180	50.5–105.6
		Av ± SD^1^	285 ± 117	0.102 ± 0.021	390 ± 37	0.191 ± 0.058	8.61 ± 0.35	240 ± 13	0.018 ± 0.008	7214 ± 148	527 ± 18	2102 ± 139	16,702 ± 537	0.125 ± 0.009	62.18 ± 4.73
		Av^2^	–	–	366	–	–	387	–	8758	832	2531	13,486	–	132.5
		Range^3^	–	–	–	0.43–6.56	7.69–28.21	–	0.010–0.059	–	–	–	–	0.07–3.29	71.3–243.1
		Av ± SD^3^	–	–	–	1.99 ± 1.46	18.71 ± 5.26	–	0.023 ± 0.010	–	–	–	–	1.93 ± 0.80	139.8 ± 41.9
		Av^6^	–	0.059	–	0.23	–	–	0.010	–	–	–	–	0.19	–

– no data

^1^This study (2017)/Poland (dry weight)

^2^Data from https://ndb.nal.usda.gov/; (wet weight calculated to dry weight)

^3^Kołacz et al. (2003)/Poland (wet weight calculated to dry weight)

^4^Oyaro et al. (2007)/Nairobi, Kenya (dry weight)

^5^Sikora (1994)/Poland (wet weight calculated to dry weight)

^6^Szkoda and Żmudzki (2002)/Poland (wet weight calculated to dry weight)

Group 3 included elements found in concentrations of several ppm: Mn, Cr, Mo, Ni, and Sn (Fig. 2c). Some of these elements (e.g., Mn, Cr) are involved in carbohydrate or calcium metabolism; others (e.g., Mo, Sn) play a role in oxidation–reduction processes. The highest mean Mn content was found in the turkey liver (11.85 mg kg^−1^ DW); Cr and Sn—in the chicken liver (3.39 and 1.53 mg kg^−1^ DW, respectively); Mo—in the pig liver (2.78 mg kg^−1^ DW); and Ni—in the goat liver (2.21 mg kg^−1^ DW). The lowest mean Mn, Cr, Mo, Ni, and Sn contents were found (respectively, in mg kg^−1^ DW) in the following: chicken (6.42), duck (2.48), rooster (1.45), turkey (1.90), and pig livers (1.23).

Group 4 included those microelements that are considered as having no benefit with regard to growth and development: Cd, Pb, As, Hg, and Co. In high concentrations, these elements can be highly toxic or even carcinogenic. The highest Cd, Pb, As, Hg, and Co contents were found (respectively, in mg kg^−1^ DW) in the following: rooster (0.896), duck (0.650), pig (0.163), and turkey livers (0.051 and 0.100) (Fig. 2d). The lowest values were found (respectively, in mg∙kg^−1^ DW) in the following: chicken (0.191), duck (0.125), rooster (0.043), turkey (0.018), and pig livers (0.033).

These results were higher than those reported by Szkoda and Żmudzki (2002) for pigs and rabbits (Table 6). The present Cd levels were lower than those reported by Kołacz et al. (2003) in their analysis of the chicken liver. The Pb content in the pig, cow, chicken, and rabbit livers found in the present study was similar to that reported by Szkoda and Żmudzki (2002), while that found in the duck liver was higher (Table 6). With regard to As and Hg, Szkoda and Żmudzki (2002) found considerably lower values for the pig, cow, rabbit, and chicken livers (approx. two to five times lower).

In the studied farm animal population, statistical analyses of age-related differences in element content would be pointless, as the groups were small and did not differ greatly in terms of age.

### Health risk involved in consumption of the animal liver

Consumption of food not only supplies the necessary energy but also fulfills the body’s demand for macro- and microelements. The WHO publishes the recommended intakes of these substances, which ensure healthy development while preventing any toxic effects resulting from excesses or deficiencies. National legislations also contain recommendations on the allowed content of selected elements (mainly trace elements) in a variety of food products (including the liver). Thus, the Polish Health Ministry Regulation of 2003 and the Commission Regulation (EC No. 1881/2006) stipulate the maximum allowed content of particularly harmful elements in the animal liver, which amounts to 0.50 mg kg^−1^ FW for As, Cd, and Pb, and 0.05 mg kg^−1^ FW for Hg. Comparisons between the test results and the maximum content limits showed that, in the case of wild animals, the regulatory limits were exceeded in 18% (for Cd and Cu) and 9% (for Hg) of the liver samples analyzed. The results for farm animal liver samples are much better, as none of these elements exceeded the allowed limits (Table 2). Among macroelements, maximum content is only regulated for Cu and Zn and amounts to 10 and 150 mg kg^−1^ FW, respectively. Based on the obtained results, 18% of wild animals and 9% of farm animals have excessive levels of Cu, but all samples were within the regulatory limits for Zn.

### RDI

For the purpose of assessing the health risk involved in human consumption of animal products, we used 100 g as the daily animal liver consumption, based on the FAO/WHO expert committee report on veterinary drug residues (EC No. 782/2018). Provisional Tolerable Weekly Intake (PTWI) and other selected intake indicators (including ADI, MRL, RDI, TDI—abbreviations are explained below Table 7) for the selected macro- and microelements based on FAO/WHO recommendations are shown in Table 7. The table also includes the RfD (reference dose) values used in HQ calculations (see “Study methods,” formula 2 above). The collected data were used to propose a Recommended Daily Intake (RDI) value for each of the elements analyzed. A comparison between the calculated RDI for children (BW = 20 kg) and adults (BW = 70 kg) and the levels of selected elements found in the animal liver (converted to fresh weight using formula 1) demonstrated that:

1. In the samples from wild animals, the calculated RDI values for children were exceeded in one sample with regard to Pb, Hg, As, and Zn; in three samples with regard to Cu; in four—P; in five—Al; in six—Cd, and in seven—Fe. The levels of other elements, i.e., Zn, Co, Ni, and Mo, did not exceed the RDI.
  Results for farm animal liver samples (*n* = 22) were somewhat better. RDI values were not exceeded for Sn, Co, Ni, Mo, Pb, Hg, As, or Zn. Cd content exceeded the limit in just 1 sample, Fe—in four samples, Cu—five, Al—eight, and P—nine samples.
2. The RDI values for adults were not exceeded in samples from farm and wild animals with regard to: Ca, K, Hg, Mg, Mo, Na, P, Ni, Mn, Zn, As, Co, and Sn. In one sample from a wild animal (boar), the RDI for Pb and Cd was exceeded. The RDI for Cu was exceeded in two liver samples, boar and deer, and the RDI for Fe was exceeded in seven boar liver samples. In the farm animal liver samples, RDI for Cd was exceeded in 1 case (turkey), Al—in three cases (cow, rabbit, chicken), Cu—in three (goat, duck, rabbit), and Fe—in four (got, duck, pig, turkey).
3. Cr levels were the most alarming, as they exceeded the RDI both for children and for adults in all the analyzed liver samples, collected from wild as well as farm animals.

**Table 7:** Recommended Daily Intake (RDI) for children and adults of macro- and microelements

Element	PTWI^1^ (mg/kg bw)	Recommendation	RfD^6^	RDI (mg/day) for:	
				Adults (bw = 70 kg)	Children (bw = 20 kg)
Ca	N.S.	1000 mg/day for adults, 1300 mg/day for youth, post-menopausal women, the elderly^7^	N.S.	1200	1000
K	N.S.	4700 mg/day for adults, 2400–3700 mg/day for children^7^	N.S.	4700	3100
Mg	N.S.	265–420 mg/day for adults, 240–410 mg per day for youth, 80–130 mg for children (6 years old) ^7^	N.S.	400	130
Na	N.S.	1300–1500 mg/day for adults, 750–1000 mg/day for children^7^	N.S.	1200	1000
P	N.S.	700 mg/day for adults, 1250 mg/day for youth, 500 mg for children (6 years old)^7^	N.S.	700	500
Al	2^1^	MRL 1 mg/kg/day^5^	1.00E+00	20	5.7
As	0.015^2^	Best estimate 0.1–3 μg/kg bw/day^1^ MRL 0.005 mg/kg/day^5^	3.00E−04	0.15	0.04
Cd	0.007^3^	PTMI 25 μg/kg bw/month^1^ MRL 0.0005 mg/kg/day^5^	1.00E−03	0.07	0.02
Co	N.S.	RDI 1 μg/kg bw/day^4^ MRL 0.01 mg/kg/day^5^	2.00E−02	0.07	0.02
Cr	N.S.	ADI 5 μg/kg bw/day^4^ RDI 35 μg^4^ MRL 0.0009 mg/kg/day^5^	3.00E−03	0.035	0.01
Cu	N.S.	PMTDI 0.5 mg/kg bw/day^1^ ADI 2–3 mg/day for adults; 0.5–0.7 mg/day for infants^1^ MRL 0.01 mg/kg/day^5^	4.00E−02	0.9	0.4
Fe	N.S.	PMTDI 0.8 mg/kg bw per day,^1^ ADI 17 mg/day (males, age 20–34 years) 9–12 mg/day (females), 7–15 mg/day^7^	3.00E−01	10	10
Hg	0.004^1^	MRL 0.002 mg/kg/day^5^	3.00E−04	0.035	0.01
Mn	N.S.	RDI 2–3 mg/day^4^ TDI 8–9 mg/day (adults)^4^	2.40E−02	210	60
Mo	N.S.	RDI for adults 0.075–0.250 mg/kg bw/day^4^ MRL 0.008 mg/kg/day^5^	5.00E−03	17.5	5
Ni	0.035^1^	TDI 5 μg/kg bw/day^4^	2.00E−02	0.35	0.1
Pb	0.025^1^	RDI for adults 0.02–3 μg/kg bw/day (mean) and for children 0.03 to 9 μg/kg bw/day (mean)^1^	3.50E−03	0.21	0.07
Sn	14^1^	MRL 0.3 mg/kg/day^5^	N.S.	140	40
Zn	N.S.	PMTDI 0.3–1 mg/kg bw/day^1^ RDI 14–20 mg/day^1^ MRL 0.3 mg/kg/day^5^	3.00E−01	7	5

*N.S.* not specified, *RDI* Recommended Daily Intake, *TDI* Tolerable Daily Intake, *ADI* Acceptable Daily Intake, *PTWI* Provisional Tolerable Weekly Intake, *PTMI* Provisional Tolerable Monthly Intake, *PTMI* Provisional Tolerable Monthly Intake

^1^http://apps.who.int/food-additives-contaminants-jecfa-database/chemical.aspx?chemID = 3511

^2^Recommendation from TRS 776-JECFA 33/27 (1988)

^3^Recommendation from TRS 930-JECFA 64/26 (2005)

^4^Kabata-Pendias and Szteke (2012) data for adults

^5^MRL—minimal risk levels, data from https://www.atsdr.cdc.gov

^6^Data from https://cfpub.epa.gov/ncea/iris2 for oral exposure

^7^Data from IŻŻ—National Food and Nutrition Institute (Poland)

### HQ

Non-carcinogenic health risk was analyzed for the following elements: Al, As, Cd, Co, Cr, Cu, Fe, Hg, Mn, Mo, Ni, Pb, and Zn, due to the availability of RfD values that could be used in hazard quotient (HQ_ing_) calculation. HQ_ing_ was calculated for children and adults with an assumed F_IR_ = 100 g (Table 8).

**Table 8:** HQ, risk, and HI values calculated for daily and weekly intakes of the wild and farm animals’ liver

Elements	HQ_ing_ or risk for consumption liver from							
	Wild animals				Farm animals			
	Daily		Weekly		Daily		Weekly	
	Adults	Children	Adults	Children	Adults	Children	Adults	Children
Al	9.57E−02	3.35E−01	1.36E−02	4.77E−02	1.16E−01	4.05E−01	1.65E−02	5.77E−02
As—non-cancer	2.38E−01	8.33E−01	3.39E−02	1.19E−01	1.43E−01	5.00E−01	2.04E−02	7.12E−02
Cd—non-cancer	5.00E−01	*1.75E+00*	7.12E−02	2.49E−01	1.43E−01	5.00E−01	2.04E−02	7.12E−02
Co—non-cancer	1.43E−03	5.00E−03	2.04E−04	7.12E−04	1.43E−03	5.00E−03	2.04E−04	7.12E−04
Cr—non-cancer	4.00E−01	*1.40E+00*	5.70E−02	1.99E−01	4.29E−01	*1.50E+00*	6.11E−02	2.14E−01
Cu	2.14E−01	7.50E−01	3.05E−02	1.07E−01	2.50E−01	8.75E−01	3.56E−02	1.25E−01
Fe	6.38E−01	*2.23E+00*	9.09E−02	3.18E−01	5.81E−01	*2.03E+00*	8.28E−02	2.90E−01
Hg	9.52E−02	3.33E−01	1.36E−02	4.75E−02	4.76E−02	1.67E−01	6.78E−03	2.37E−02
Mn	6.21E−02	2.17E−01	8.85E−03	3.10E−02	6.21E−02	2.17E−01	8.85E−03	3.10E−02
Mo	2.29E−01	8.00E−01	3.26E−02	1.14E−01	1.74E−01	6.10E−01	2.48E−02	8.69E−02
Ni—non-cancer	4.93E−02	1.73E−01	7.02E−03	2.46E−02	4.50E−02	1.58E−01	6.41E−03	2.24E−02
Pb	5.71E−02	2.00E−01	8.14E−03	2.85E−02	2.45E−02	8.57E−02	3.49E−03	1.22E−02
Zn	1.29E−01	4.50E−01	1.83E−02	6.41E−02	1.00E−01	3.50E−01	1.42E−02	4.99E−02
HI (non-cancer)	*2.71E+00*	*9.48E+00*	3.86E−01	*1.35E+00*	*2.12E+00*	*7.40E+00*	3.02E−01	*1.06E+00*
As—cancer	9.18E−06	3.21E−05	1.31E−06	4.58E−06	5.51E−06	1.93E−05	7.85E−07	2.75E−06
Risk (cancer)	9.18E−06	3.21E−05	1.31E−06	4.58E−06	5.51E−06	1.93E−05	7.85E−07	2.75E−06

Italicized values: HQ_ing_, HI > 1

For children and adults eating liver from wild animals, either once daily or once weekly, the following sequence of HQ_ing_ values was obtained, in descending order:

$$ \mathrm{Fe}>\mathrm{Cd}>\mathrm{Cr}>\mathrm{As}>\mathrm{Mo}>\mathrm{Cu}>\mathrm{Zn}>\mathrm{Al}>\mathrm{Hg}>\mathrm{Mn}>\mathrm{Pb}>\mathrm{Ni}>\mathrm{Co}. $$

With regard to the liver from farm animals, the descending HQ_ing_ value sequence for children and adults was different:

$$ \mathrm{Fe}>\mathrm{Cr}>\mathrm{Cu}>\mathrm{Mo}>\mathrm{As}=\mathrm{Cd}>\mathrm{Al}>\mathrm{Zn}>\mathrm{Mn}>\mathrm{Hg}>\mathrm{Ni}>\mathrm{Pb}>\mathrm{Co}. $$

The differences between the two sequences above result from different content of particular metals (especially Cd and Cu) in the two sample groups.

Based on the specific HQ_ing_ values calculated for children, we found HQ_ing_ values > 1, which indicate a possible health risk, only for daily consumption of the liver from wild animals, due to Fe (2.23E+00), Cd (1.75E+00), and Cr (1.40E+00) content. With the farm animal liver, the only risks were related to Fe (2.03E+00) and Cr (1.50E+00) content. With once per week consumption of the liver, the HQ_ing_ values for all the analyzed elements were below 1, which indicates no health risk. For adults, all HQ_ing_ values were below 1, both for daily and weekly consumption of the liver and for wild and farm animals. This warrants the conclusion that the product does not pose a health risk.

While the high Fe content in the animal liver is not problematic (indicating instead that the liver is an important source of highly absorbable Fe). Iron overload may occur but only when the element is delivered in supplements. With iron delivered in food, the organism carefully regulates its levels, assimilating this microelement according to the current needs. Any excess of this element is safely removed (it is not assimilated). The most frequent reason for elevated iron levels is a genetic predisposition for accumulation of iron, i.e., hemochromatosis. It is usually caused by a mutation of the *HFE* gene. The health risk associated with high Cd and Cr content can be alarming.

Cd accumulates in the human body throughout the life, due to the element’s long half-life (10–33 years). Kabata-Pendias and Szteke (2012) state that up to 15% of adsorbed Cd can accumulate in the liver. The metal inhibits phosphatase and supersedes Zn, which is required for healthy function of the body. Excess Cd impairs kidney function, Ca metabolism, and vitamin B12 transformation, leading to anemia and hypertension. The accumulation of Cd is particularly harmful in young individuals, causing irreversible alterations to the central nervous system. This is why the element is considered a group 1 carcinogen.

Cr was another element with a HQ_ing_ > 1. It is needed for human and animal health, and has two forms: Cr^3+^ and Cr^6+^. Cr plays an important role in protein and lipid metabolism, as well as in the action of insulin. In food, it is mainly present as Cr^3+^, though this does not apply to the liver. Cr content in the body decreases with age, and the decrease is particularly marked in the liver. Therefore, the element is often included in the so-called geriatric supplements.

### Hazard and risk indexes

The final hazard index (HI) values, which are the sum of all HQ_ing_ values calculated for children and adults, considering daily or weekly consumption, are shown in Table 8. The HI values for children are approx. 3.5 times higher on average than HI values for adults. The HI values for children and adults with the same exposure frequency, but different sources of the liver (farm or wild animals), differ by approx. 30%, which is due to the different element content in the liver of wild and farm animals.

The HI for children (with daily or weekly consumption of the liver from wild or farm animals) and for adults (only with daily consumption, but either source of the liver) is greater than 1, which may indicate a possible adverse health impact. No such risk was found for adults eating liver from wild or farm animals once weekly (Table 8).

The HI values for element content in the farm animal liver were lower than those for the wild animal liver, with regard to consumption by both children and adults. In the context of the analyzed macro- and microelement contents, this may indicate that the farm animal liver is “safer” for human consumption.

With daily animal liver consumption in the case of children, the risk index was 3.21E−05 and 1.93E−05 for wild and farm animals, respectively. These values are close to the limit considered safe (10E−05). As for the scenario assuming animal liver consumption once weekly and once daily in the case of adults, the risk index ranged between 7.85E−07 and 9.18E−06. Since these values are below the permissible limit, this situation can be deemed satisfactory.

## Conclusions

Based on our analysis of macro- and microelement contents in 33 liver samples collected from wild animals (boar, deer) and farm animals (chicken, cow, duck, turkey, goat), the following conclusions can be formulated:

1. No significant differences were found in the macroelement levels analyzed (Al, Ca, Cu, Fe, K, Mg, Mn, Na, P, S, and Zn) between the livers from wild and farm animals. The farm animal liver contained more Al, Cu, K, Mg, and Na (the difference ranged between 9 and 23%). Mn and P levels were found to be nearly identical in these two animal groups. Slightly lower results were obtained for farm animals than for wild animals with regard to content of Zn, Ca, and Fe.
2. With regard to microelements (As, Cd, Co, Cr, Hg, Mo, Ni, Pb, and Sn), farm animal liver had a higher mean content of Cr and Sn only. The mean levels of the remaining elements were between 4 and 70% lower than in wild animals.
3. Statistically, the differences in element contents between the livers from wild and farm animals were not significant (at *p* = 0.05), with the exception of Fe, K, Mg, Cd, Hg, Mo, and Pb.
4. In the samples from farm animals, there was a considerably larger dispersion of macroelement levels, and the highest variability in the entire material was found for the concentrations of: Hg, Cu, Fe, Al, and Cd.
5. The liver of female boars contained less Al, K, Mg, As, Pb, Zn, and Hg but more Cr, Na, Sn, Ca, Fe, Mo, Ni, Mn, S, Co, Cu, and Cd. P concentrations were nearly identical in both sexes. Very high variability was found for Al and Hg in males and for Cd and Cu in females. With a significance threshold set at *p* = 0.05, the calculated differences in levels of the analyzed elements between the livers from male and female boars were not significant.
6. An analysis of correlations between element content and age in wild animals showed that Pb and Al contents increase with age, while Na and Cr contents decrease significantly. For the remaining elements, no statistically significant correlations were found.
7. Comparisons between the test results and the maximum limits allowed by law showed that, in the case of wild animals, the regulatory limits were exceeded in 18% (for Cd and Cu) and 9% (for Hg) of the liver samples analyzed. The results for farm animal liver samples are much better, as none of these elements exceeded the allowed limits.
8. The HI values for the liver from farm animals were lower than for wild animals, with regard to consumption by both children and adults. In the context of the analyzed macro- and microelement contents, this may indicate that the farm animal liver is “safer” for human consumption.
9. Based on the HI values calculated, it seems recommendable that consumption of the liver (preferably from farm animals) by children be limited to once weekly.
10. For adults, the liver can be a valuable source of elements such as Zn, Fe, and Cr, which may warrant more frequent consumption.
